# Association of Obesity and Kidney Function Decline among Non-Diabetic Adults with eGFR > 60 ml/min/1.73m^2^: Results from the Multi-Ethnic Study of Atherosclerosis (MESA)

**DOI:** 10.4236/ojemd.2013.32016

**Published:** 2013-05-23

**Authors:** Anna Malkina, Ronit Katz, Michael G. Shlipak, Joachim H. Ix, Ian H. de Boer, Mark J. Sarnak, Matthew Allison, Holly J. Kramer, Julie Lin, David Siscovick, Carmen A. Peralta

**Affiliations:** 1 University of California, San Francisco, USA; 2 University of Washington, Seattle, USA; 3 San Francisco Veterans Affairs Medical Center, San Francisco, USA; 4 University of California, San Diego, USA; 5 Tufts Medical Center, Boston, USA; 6 Loyola University Medical Center, Maywood, USA; 7 Harvard University, Cambridge, USA

**Keywords:** Kidney Function Decline, MESA, Obesity, Waist Circumference, Waist-to-Hip Ratio

## Abstract

**Background:**

Obesity is associated with higher end-stage renal disease incidence, but associations with earlier forms of kidney disease remain incompletely characterized.

**Methods:**

We studied the association of body mass index (BMI), waist circumference (WC), and waist-to-hip ratio (WHR) with rapid kidney function decline and incident chronic kidney disease in 4573 non-diabetic adults with eGFR ≥ 60 ml/min/1.73m^2^ at baseline from longitudinal Multi-Ethnic Study of Atherosclerosis cohort. Kidney function was estimated by creatinine and cystatin C. Multivariate analysis was adjusted for age, race, baseline eGFR, and hypertension.

**Results:**

Mean age was 60 years old, BMI 28 kg/m^2^, baseline eGFR_Cr_ 82 and eGFR_Cys_ 95 ml/min/1.73m^2^. Over 5 years of follow up, 25% experienced rapid decline in renal function by eGFR_Cr_ and 22% by eGFR_Cys_. Incident chronic kidney disease (CKD) developed in 3.3% by eGFR_Cys_, 11% by eGFR_Cr_, and 2.4% by both makers. Compared to persons with BMI < 25, overweight (BMI 25 – 30) persons had the lowest risk of rapid decline by eGFR_Cr_ (0.84, 0.71 – 0.99). In contrast, higher BMI categories were associated with stepwise higher odds of rapid decline by eGFR_Cys_, but remained significant only when BMI ≥ 35 kg/m^2^ (1.87, 1.41 – 2.48). Associations of BMI with incident CKD were insignificant after adjustment. Large WC and WHR were associated with increased risk of rapid decline only by eGFR_Cys_, and of incident CKD only when defined by both filtration markers.

**Conclusions:**

Obesity may be a risk factor for kidney function decline, but associations vary by filtration marker used.

## 1. Introduction

Obesity is an epidemic in the United States, and its prevalence has risen in the last decades [1,2]. Obesity is a known risk factor for adverse health outcomes, including common kidney disease risk factors such as diabetes and hypertension [3]. However, whether or not there is an independent association between obesity and kidney disease is less well established. High body mass index (BMI) has been shown to be an independent risk factor for development of End Stage Renal Disease (ESRD) [4], but associations of obesity with earlier forms of kidney disease remain less clear. Understanding these associations may elucidate potentially modifiable risk factors to reduce the burden of chronic kidney disease (CKD) [5].

Prior studies examining the association of obesity and risk of CKD have been conflicting. For example, some studies have shown that high BMI was associated with the development of CKD, defined as sex-specified cutoffs in creatinine-based estimated glomerular filtration rate (eGFR_Cr_) around 60 ml/min/1.73m^2^, creatinine ≥1.5 mg/dL, or presence of +1 or greater proteinuria on urine dipstick [6–8]. However, other cohorts, including the Framingham Offspring Study and Cardiovascular Health Study suggest that the association of high BMI with development of CKD and rapid kidney function loss was attenuated by adjustment for presence of traditional cardiovascular disease risk factors [9,10]. Other studies have suggested that measurements of central obesity, such as waist circumference (WC) or waist to hip ratio (WHR), rather than BMI, may be more strongly and independently associated with increased risk for incident CKD, but findings have not been consistent across studies [11, 12].

Prior literature may be limited by the use of inconsistent measures of obesity, and variable laboratory definitions of CKD. Since direct measurement of GFR is not practical in routine clinical and research setting, studies vary in use of surrogate markers and calculation formulas for estimation of GFR. Most studies utilize creatinine, which is a by-product of muscle metabolism, and therefore biased by overall muscle mass [13,14]. Alternatively, cystatin C is another endogenous protein produced by nearly all cells, generated constantly irrespective of muscle mass, and has been shown to provide as accurate estimate of GFR as creatinine [15]. Furthermore, cystatin C has been shown to have stronger association with mortality and cardiovascular diseases than creatinine in older adults, and higher specificity for detection of CKD and adverse outcomes (risk of death, cardiovascular disease, heart failure, and kidney failure) in multiethnic ambulatory cohorts [16,17]. Therefore, we designed this study to investigate whether obesity is associated with rapid kidney function decline and incident CKD in a multi-ethnic cohort of non-diabetic adults with baseline eGFR > 60 ml/min/1.73m^2^. We used three anthropometric measures of obesity (BMI, WC, and WHR), and both creatinine and cystatin C as measures of kidney function.

## 2. Methods

### 2.1. Subjects

We included participants from Multi-Ethnic Study of Atherosclerosis (MESA), a prospective cohort designed to study cardiovascular disease risk in a multi-ethnic population. Details on recruitment and design have been previously published [18]. Briefly, MESA recruited 6814 men and women between the ages of 45 and 84 who were: free of cardiovascular disease, and self-identified as White, African American, Hispanic, or Chinese American. MESA recruited persons from 6 sites across the US (Baltimore City and County, Maryland; Chicago, Illinois; Forsyth County, North Carolina; Los Angeles County, California; northern Manhattan and Bronx, New York; and St. Paul, Minnesota) from July 2000 to August 2002. Participants returned for follow up exams at 18 months, 3 and 5 years. Renal function was measured at years 0, 3, and 5. The original design excluded individuals with body weight over 300 lbs. The institutional review boards at all participating centers approved the study, and all participants gave informed consent.

For these analyses, we excluded individuals with no measures of albuminuria, creatinine, or cystatin C at baseline (N = 94); those without follow up measures of either creatinine or cystatin C (N = 799); persons with baseline CKD (defined as eGFR_Cr_ < 60ml/min/1.73m^2^ based on current clinical practice guidelines [19]) (N = 742); without anthropometric measures (N = 19); and with diagnosis of diabetes at baseline (N = 587). We elected to study only non-diabetic participants as literature suggests that the physiology of obesity-related glomerulopathy may differ for persons with diabetes [20–22]. Our total sample size was 4573.

### 2.2. Primary Predictors

Anthropometric measurements (height, weight, waist and hip circumferences) were obtained at each participant’s baseline visit. Height and weight were measured with participants wearing light clothing and no shoes. BMI was calculated as weight (kg) divided by height (m^2^), and classified into 4 categories (<25, 25.0 – 29.9, 30.0 – 34.9, and ≥35.0) based on NHLBI Obesity Education Initiative 2000 guidelines [23]. BMI < 25 kg/m^2^ was used as a reference category. WC was measured in cm at the level of the umbilicus and dichotomized using categories of 88 cm for women and 102cm for men based on ATPIII 2002 guidelines [24]. WHR was dichotomized using categories of 0.85 for women and 0.9 for men based on World Health Organization 1999 guidelines [25]. The smaller WC and WHR categories were used as references. In a sensitivity analysis we also looked at WC and WHR categorized in quintiles.

### 2.3. Outcomes

Kidney function was measured by creatinine and cystatin C, with repeated measurements over 5 years. All assays were performed in frozen serum specimens that were obtained in fasting state and stored at −70°C. Serum creatinine was measured by rate reflectance spectrophotometry using thin film adaptation of the creatine amidinohydrolase method on the Vitros analyzer (Johnson & Johnson Clinical Diagnostics) at the Collaborative Studies Clinical Laboratory at Fairview University Medical Center, and calibrated to Cleveland Clinic. Cystatin C was measured by means of a particle-enhanced immunonephelometric assay (N Latex Cystatin C, Dade Behring) with a nephelometer (BNII, Dade Behring) and corrected for assay drift. We estimated GFR_Cr_ using CKD-Epi equation [26], and GFR_Cys_ using cystatin C equation [15].

Our two outcomes of interest were rapid kidney function decline and incident CKD. Rapid decline was defined as eGFR decline of >5% ml/min/year during the follow up period [27]. This cut-off was chosen because it corresponded to the top quintile of kidney function decline in our cohort, and also closely corresponds to >3 ml/min/year change in eGFR, which has been shown in prior studies to be associated with increased risk of adverse outcomes [28]. We conducted separate analyses with creatinine and cystatin C.

Incident CKD was defined as development of eGFR < 60 ml/min/1.73m^2^ and decline in eGFR > 1 ml/min/year at any of the follow up visits using creatinine, cystatin C, and both markers combined. We include a definition based on cystatin C because we have shown that it reduces misclassification and improves risk stratification of persons with CKD [17,19].

### 2.4. Covariates of Interest

Information on age, sex, and self-reported race/ethnicity was obtained using standardized questionnaires. Blood pressure measurements were obtained using the Dinamap^®^ automated blood pressure device (Dinamap Monitor Pro 100^®^). Three sequential measures were obtained and the average of the second and third measurements was recorded. Hypertension was defined as systolic pressure ≥ 140 mm Hg, diastolic pressure ≥ 90 mm Hg, or current use of antihypertensive medication. Diabetes was defined as either a fasting glucose ≥ 126 mg/dl or use of oral hypoglycemic medication or insulin. Cigarette smoking was defined as current, former, or never. High density lipoprotein (HDL) cholesterol was measured using the cholesterol oxidase cholesterol method (Roche Diagnostics). Low-density lipoprotein (LDL) cholesterol was calculated using the Friedewald equation. Urine albumin and creatinine were measured in a single morning urine sample by nephelometry and the rate Jaffe reaction, respectively, and expressed as albumin to creatinine ratio (ACR) in mg/g.

### 2.5. Statistical Analyses

First, we evaluated characteristics of MESA participants at baseline by BMI categories. We used ANOVA or Chi-Square where appropriate.

We then estimated prevalence of rapid decline by categories of BMI, WC, and WHR using creatinine and cystatin C separately. To evaluate the associations between BMI, WC, and WHR with rapid kidney function decline, we used logistic regression models. Candidate covariates included possible confounders, such as age, sex, race, and baseline eGFR; as well as potential variables in the pathophysiological pathway, such as hypertension, fasting glucose, and HDL and LDL cholesterol. We forced *a priori* variables that are known to be confounders or strong established CKD predictors (age, sex, race, baseline eGFR). Remaining variables were only included if they changed beta coefficient by >5%. Model 1 adjusted for age and baseline eGFR; and model 2 adjusted for model 1 plus sex, race, and hypertension. In a separate sensitivity analysis, we substituted hypertension with systolic blood pressure as a continuous variable due to its strong and linear association with kidney disease.

For the outcome of incident CKD we used Poisson (log-link) regression with analysis models as discussed above. For these analyses, we excluded an additional 76 participants with baseline eGFR_Cys_ < 60ml/min/1.73m^2^, with resulting sample size of 4497.

To understand whether the presence of albuminuria modified any observed associations, we excluded 232 persons with ACR ≥ 30 mg/g at baseline, and repeated our analyses.

## 3. Results

### 3.1. Baseline Characteristics

Among 4573 non-diabetic adults in MESA, mean age was 60 ± 10 years old, 48% were men, 12% were Chinese, 27% were Black, and 22% were Hispanic. Mean BMI was 28 ± 5.3 kg/m^2^, 40% were overweight (BMI 25 – 29.9 kg/m^2^), and 30% were obese (BMI ≥ 30 kg/m^2^). Mean baseline eGFR_Cr_ was 82 ± 13 and eGFR_Cys_ was 95 ± 16 ml/min/1.73m^2^. We found race differences in the prevalence of obesity, with Black participants having the highest prevalence of BMI ≥ 35.0 kg/m^2^. HDL was the lowest among obese persons, but LDL and triglycerides did not vary across the BMI categories. Persons with higher BMI were more likely to be hypertensive, but less likely to be current smokers. eGFR_Cys_ was progressively lower with increasing BMI category, but not eGFR_Cr_. Prevalence of ACR > 30 mg/g increased with rise in BMI category (Table 1).

### 3.2. Association of BMI, WC, and WHR with Rapid Kidney Function Decline

Median follow-up up time was 4.8 years. Among the participants in this study 25% (N = 1161) had rapid decline by eGFR_Cr_, and 22% (N = 988) by eGFR_Cys_. Mean absolute decline in persons with rapid decline was 4.57 ml/min/1.73m^2^ annually by eGFR_Cr_ and 6.33 ml/min/1.73m^2^ annually by eGFR_Cys_.

**Figure 1:**
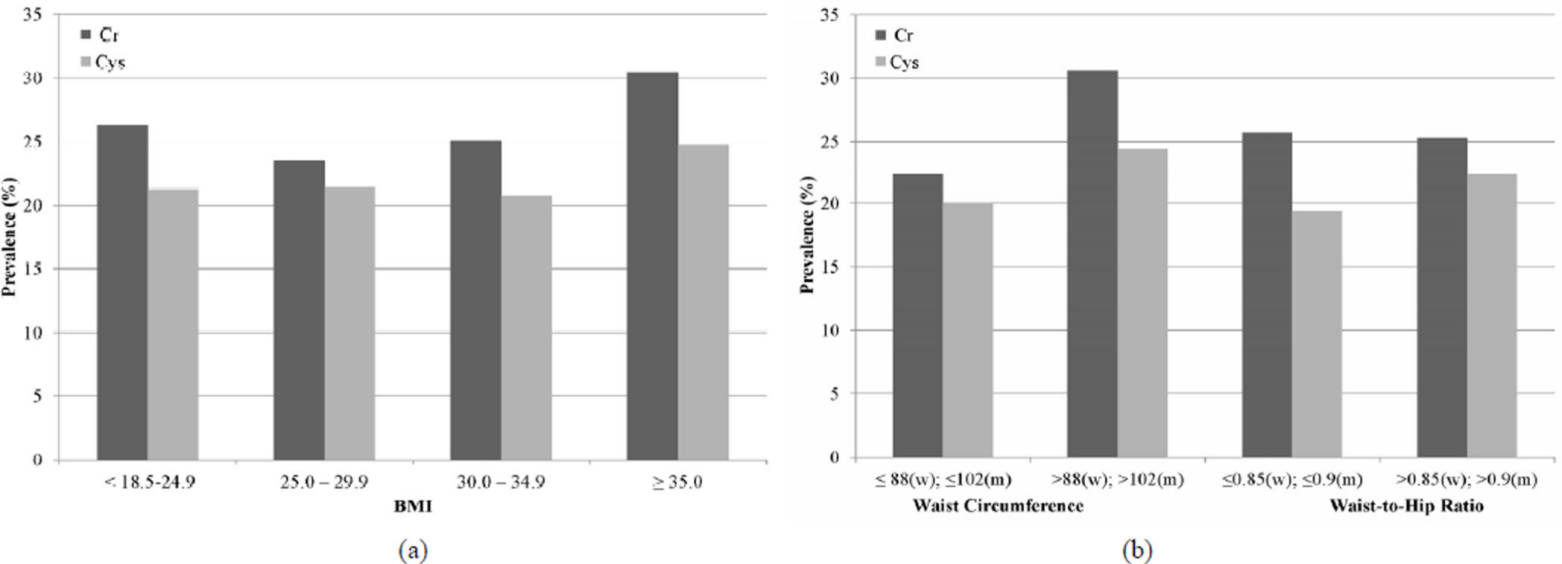
Unadjusted prevalence of rapid decline among MESA participants with GFR > 60 mL/min/1.73m^2^ estimated by creatinine (Cr) and cystatin C (Cys). (a) Body mass index; (b) Waist circumference and waist to hip ratio.

First, we estimated the prevalence of rapid decline by BMI category, using eGFR_Cr_ and eGFR_Cys_ separately. With eGFR_Cr_, we found that the association of BMI and rapid decline appeared to be U-shaped with overweight persons (BMI 25.0 – 29.9 kg/m^2^) having the lowest risk of rapid decline (Figure 1(a)).

We also studied the association of high WC and high WHR with rapid decline. In age-adjusted models, high WC was associated with rapid decline by creatinine and cystatin C measures (Figure 1(b)). However, in fully adjusted models, the association was statistically significant at 38% higher risk only with cystatin C (Table 2). High WHR was not associated with rapid decline when using eGFR_Cr_. In contrast, persons with high WHR were at 21% higher odds of rapid decline when using eGFR_Cys_ and this was moderately attenuated to 16% with full adjustment (Table 2).

In adjusted models persons in the overweight category had 16% lower odds of rapid decline compared to persons with BMI < 25 kg/m^2^. A ssociations were not statistically significant for higher BMI categories. In contrast, with eGFR_Cys_, higher BMI categories had a step-wise increase in risk of rapid decline (Figure 1(a)). After full adjustment, only persons in the highest BMI category (BMI ≥ 35 kg/m^2^) remained at statistically significant increased risk of rapid decline (Table 2).

### 3.3. Association of BMI, WC, and WHR with Incident CKD

During follow-up, incident CKD was observed in 11% (N = 505) by creatinine definition, 3.3% (N = 150) by cystatin C, and 2.4% (N = 114) by both markers. When using eGFR_Cr_ to define incident CKD, persons with BMI of 25 – 29.9 had the lowest unadjusted rate of incident CKD, but this finding was not statistically significant in multivariate models. When using eGFR_Cys_, higher BMI was associated with a stepwise increase in age-adjusted rates of incident CKD, and this was attenuated after adjustment. When requiring both markers to define CKD, persons in the highest BMI categories appeared to have almost double the incidence of CKD compared to persons with normal BMI in age-adjusted models, but these findings were not significant after full adjustment (Table 3).

In contrast, larger waist circumference was associated with higher incidence of CKD in age adjusted models using all three definitions of incident CKD. Though this association was attenuated after adjustment when using eGFR_Cr_ or eGFR_Cys_, it remained significant when using both markers to define CKD (IRR 1.72, CI 1.07 to 2.77) (Table 3). High WHR was not associated with incident CKD (Table 3).

### 3.4. Sensitivity Analyses

We repeated our analyses after exclusion of 232 persons with ACR > 30 mg/g at bas eline, as this urinary marker may represent early kidney impairment, and has been associated with all-cause mortality and incident ESRD [28]. Our findings were not materially different. For example, when using eGFR_Cys_, higher BMI was associated with higher odds of rapid decline, but this was only statistically significant for BMI of ≥35.0 kg/m^2^ with OR 1.72 (1.28, 2.32). Similarly, the eGFR_Cr_ model demonstrated U-shaped association, with overweight persons (BMI 25.0 – 29.9 kg/m^2^) having the lowest risk of rapid decline with OR 0.84 (0.71, 1.00).

Similarly, results were not materially different when modeling WC and WHR in quintiles. For example, for highest quintile of WC > 109 cm and WHR > 0.99, the odds of rapid eGFR_Cys_ after full adjustment were 1.42 (1.10, 1.83) and 1.27 (0.97, 1.65) respectively. Finally, results were not materially different when we used systolic blood pressure as a continuous variable.

## 4. Discussion

In this large multi-ethnic cohort of non-diabetic adults with eGFR_Cr_ > 60 ml/min/1.73m^2^ at baseline, we found that BMI was associated with rapid kidney function decline, but the strength and shape of association varied by the filtration marker used. Using eGFR_Cr_, the overweight group had the lowest risk of rapid decline compared to those with BMI < 25 kg/m^2^, whereas risk was not different for persons in higher BMI categories. In contrast, when using eGFR_Cys_, higher BMI categories were incrementally associated with higher risk of rapid kidney function decline. Central measures of adiposity, WC and WHR, were associated with rapid decline when using eGFR_Cys_. Associations of BMI, WC, and WHR with incident CKD also varied by filtration marker used. Persons in the overweight group also had the lowest rates of incident CKD, but associations of BMI with incident CKD were largely attenuated after adjustment using all definitions. Larger WC was consistently associated with incident CKD, and remained significant only when using a combined creatinine and cystatin C definition. WHR was not significantly associated with incident CKD in these analyses. Our findings suggest that obesity, particularly central obesity, may be a modifiable risk factor for kidney function decline.

Prior literature has shown that obesity is associated with incident ESRD [4,29,30]. The longitudinal association of obesity and less severe forms of kidney disease is less well established. For example, while high BMI has been associated with incident CKD in some studies [6–8], other reports have been null [31], or are confounded by cardiovascular disease risk factors [9,10]. Some studies are limited by cross-sectional designs, self report of BMI, inconsistent definitions of incident CKD, and lack of multi-ethnic representation. In addition, most studies use creatinine to estimate glomerular filtration rate, which is known to be biased by muscle mass, and influenced by age and muscle-wasting co-morbidities [13,14]. For example, low body muscle mass, as modeled by urinary creatinine excretion rate, has been associated with increased mortality independent of anthropometric measures, eGFR, physical fitness, and traditional coronary artery disease risk factors [14]. BMI has also been associated with cystatin C in cross-sectional analyses [32–34]. Our findings expand on this literature to show that there is an association of BMI with longitudinal kidney function decline among non-diabetics, but that these associations may vary depending on the filtration marker used. Future studies will be required to disentangle non-GFR determinants of creatinine and cystatin C that may explain these differences.

Our finding that central obesity (as measured by WC and WHR) is associated with renal function decline is noteworthy. Other investigators have proposed that central measures of adiposity may be more informative for renal risk than BMI [11,12]. Furthermore, findings from the PREVEND study suggest that central obesity is associated with higher prevalence of low GFR and microalbuminuria, independent of BMI [35]. Taken together, our findings suggest that in order to understand the effect of adiposity on kidney function, several anthropometric and glomerular filtration markers should be studied concurrently.

The mechanisms by which obesity may be associated with kidney function decline are not well understood. This could be mediated via several hemodynamic, hormonal, and inflammatory processes. Obesity is associated with insulin resistance, hyperinsulinemia, and activation of renin-angiotensin-aldosterone system, all of which contribute to systemic and intraglomerular hypertension, leading to structural glomerular damage [20]. In addition to hemodynamic stress, these pathophysiologic pathways entail generation of reactive oxygen species and alteration of glomerular basement membrane composition, leading to podocyte apoptosis [20]. Furthermore, the adipose tissue, especially the visceral component, is also an active endocrine organ, generating several inflammatory cytokines (such as CRP, TNF-α, IL-6, and angiotensin II), which further exacerbate oxidative and nitrosative stress [20]. Additionally, higher leptin concentration, mainly produced by the adipose tissue, affects endothelial cells in a paracrine fashion, ultimately causing glomerulosclerosis and proteinuria [36].

To our knowledge this study is the first to study the associations of obesity with kidney disease using both creatinine and cystatin C in a longitudinal follow-up of non-diabetic adults with preserved eGFR. The strength of our study is the size and diversity of the cohort in terms of sex, age, race, and geographic sampling across six sites in the United States. Furthermore, the prevalence and racial distribution of obesity in this cohort are representative of the national data [1]. Additionally, this study collected objective measurements of three anthropometric markers, and serial measures of renal function using both creatinine and cystatin C. We are limited by a relatively short follow-up period with relatively few incident CKD cases in a healthy cohort at baseline, which may bias our results toward the null. The original design of MESA cohort also excluded persons with weight over 300 lbs, who may have the strongest association of obesity and decline in renal function. Finally, we are limited by use of indirect measures of GFR since direct measures of GFR are not practical in large epidemiologic studies. However, we believe that using two filtration markers with different non-GFR determinants may improve our ability to understand the associations of obesity and kidney disease.

In summary, our findings suggest that obesity may be an important risk factor for rapid kidney function decline and incident CKD. Given the severity of the obesity epidemic in the US, this may be a meaningful modifiable risk factor for CKD. Future studies should focus on composition of excess weight (adipose tissue vs. muscle) and its distribution within the body (central vs. peripheral) with their association with renal outcomes to further elucidate these associations.

## Figures and Tables

**Table 1:** Characteristics of non-diabetic MESA participants with eGFR > 60 ml/min/1.73m^2^ at baseline by body mass index.

Characteristics	Body mass index (kg/m^2^)			

	<25	25.0 – 29.9	30.0 – 34.9	≥35.0
**N**	1397	1826	891	459
**Age** (years)	60 (10)	61 (10)	59 (9)	58 (9)
**Men**	615 (44%)	986 (54%)	444 (50%)	118 (26%)
**Race**				
White	635 (46%)	755 (41%)	335 (38%)	150 (33%)
Chinese	362 (26%)	161 (9%)	19 (2%)	2 (0.4%)
Black	228 (16%)	463 (25%)	297 (33%)	198 (43%)
Hispanic	172 (12%)	447 (25%)	240 (27%)	109 (24%)
**Waist circumference** (cm)				
Female	81 (8)	94 (8)	105 (9)	120 (13)
Male	87 (7)	98 (6)	109 (6)	124 (8)
**Waist to hip circumference ratio**				
Female	0.85 (0.08)	0.89 (0.08)	0.92 (0.07)	0.94 (0.08)
Male	0.91 (0.06)	0.96 (0.06)	0.98 (0.05)	1.01 (0.05)
**Total cholesterol** (mg/dL)	195 (33)	195 (35)	194 (36)	195 (34)
**HDL** (mg/dL)	58 (17)	50 (13)	47 (13)	49 (12)
**LDL** (mg/dL)	116 (31)	119 (31)	118 (30)	120 (30)
**Lipid-lowering medications**	126 (9%)	295 (16%)	127 (14%)	66 (14%)
**Systolic blood pressure** (mmHg)	119 (21)	125 (19)	127 (20)	130 (21)
**Diastolic blood pressure** (mmHg)	70 (10)	73 (10)	74 (10)	72 (10)
**Hypertension**	372 (27%)	703 (39%)	406 (46%)	240 (52%)
**Hypertension medications**	260 (19%)	561 (31%)	324 (36%)	202 (44%)
**Smoking**				
Never	749 (54%)	887 (49%)	430 (48%)	218 (48%)
Former	461 (33%)	685 (38%)	337 (38%)	189 (41%)
Current	183 (13%)	251 (14%)	121 (14%)	51 (11%)
**eGFR_Cr_ at baseline** (ml/min/1.73m^2^)	82 (12)	81 (13)	81 (13)	83 (14)
**eGFR_Cys_ at baseline** (ml/min/1.73m^2^)	100 (16)	96 (16)	92 (16)	89 (16)
**ACR > 30 mg/g**	45 (3%)	86 (5%)	64 (7%)	37 (8%)

**Table 2:** Associations of obesity measures with rapid kidney function decline among non-diabetic MESA participants with eGFR > 60 mL/min/1.73m^2^.

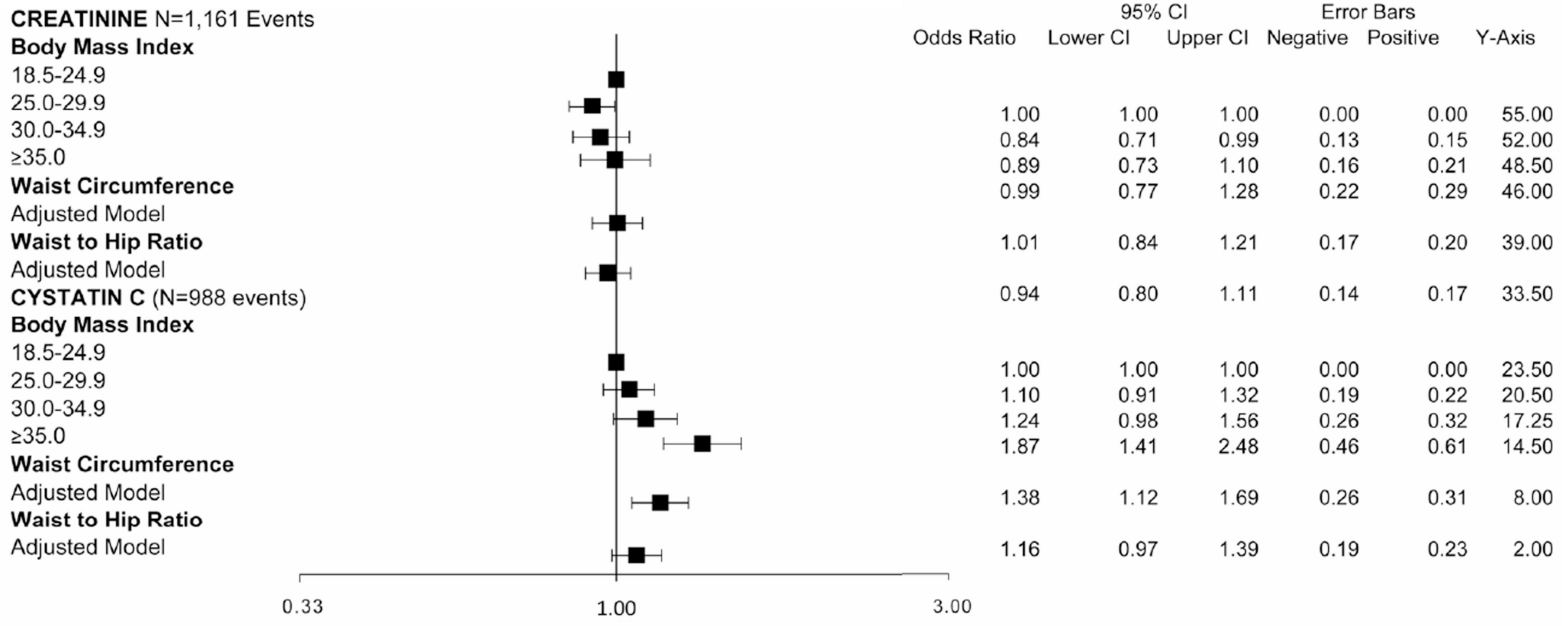

* WC: >88 (women); >102 (men); WHR: >0.85 (women); >0.90 (men).

**Table 3:** Associations of obesity measures with incident CKD among non-diabetic MESA participants with eGFR > 60 mL/min/1.73m^2^ at baseline.

	/N with incident CKD	Unadjusted rate/year	Incidence rate ratio (95% confidence interval)	

			Age-adjusted	Adjusted^a^
**Incident CKD by eGFR creatinine**				

**Body mass index** (kg/m^2^)				
<25	147	2.3	1.00 (Reference)	1.00 (Reference)
25.0 – 29.9	194	2.2	0.99 (0.81, 1.20)	0.90 (0.74, 1.10)
30.0 – 34.9	108	2.6	1.30 (1.04, 1.64)	1.06 (0.84, 1.33)
≥35.0	56	2.6	1.44 (1.08, 1.91)	1.18 (0.89, 1.57)
**Waist circumference**				
≤88 (w); ≤102 (m)	276	2.0	1.00 (Reference)	1.00 (Reference)
>88 (w); >102 (m)	229	3.0	1.40 (1.20, 1.64)	1.17 (0.94, 1.46)
**Waist to hip ratio**				
≤0.85 (w); ≤0.9 (m)	109	1.9	1.00 (Reference)	1.00 (Reference)
>0.85 (w); >0.9 (m)	396	2.5	0.97 (0.80, 1.18)	1.02 (0.84, 1.23)

**Incident CKD by eGFR cystatin C**				

**Body mass index** (kg/m^2^)				
<25	32	0.5	1.00 (Reference)	1.00 (Reference)
25.0 – 29.9	62	0.7	1.48 (0.98, 2.24)	0.95 (0.62, 1.45)
30.0 – 34.9	35	0.9	2.16 (1.35, 3.45)	0.91 (0.56, 1.47)
≥35.0	21	1.0	2.79 (1.63, 4.78)	0.85 (0.48, 1.49)
**Waist circumference**				
≤88 (w); ≤102 (m)	71	0.5	1.00 (Reference)	1.00 (Reference)
>88 (w); >102 (m)	79	1.0	1.88 (1.38, 2.56)	1.28 (0.82, 2.01)
**Waist to hip ratio**				
≤0.85 (w); ≤0.9 (m)	21	0.4	1.00 (Reference)	1.00 (Reference)
>0.85 (w); >0.9 (m)	129	0.8	1.58 (1.01, 2.49)	1.12 (0.71, 1.74)

**Incident CKD by eGFR creatinine and eGFR cystatin C**				

**Body mass index** (kg/m^2^)				
<25	27	0.4	1.00 (Reference)	1.00 (Reference)
25.0 – 29.9	45	0.5	1.25 (0.78, 1.99)	0.87 (0.53, 1.43)
30.0 – 34.9	30	0.7	2.12 (1.27, 3.55)	1.03 (0.60, 1.76)
≥35.0	12	0.6	1.86 (0.95, 3.65)	0.76 (0.38, 1.52)
**Waist circumference**				
≤88 (w); ≤102 (m)	58	0.4	1.00 (Reference)	1.00 (Reference)
>88 (w); >102 (m)	56	0.7	1.62 (1.14, 2.32)	1.72 (1.07, 2.77)
**Waist to hip ratio**				
≤0.85 (w); ≤0.9 (m)	16	0.3	1.00 (Reference)	1.00 (Reference)
>0.85 (w); >0.9 (m)	98	0.6	1.50 (0.89, 2.53)	1.11 (0.66, 1.88)

a Adjusted for age, sex, race, baseline eGFR, and hypertension.
